# The effects of a subsequent jump on the knee abduction angle during the early landing phase

**DOI:** 10.1186/s12891-018-2291-4

**Published:** 2018-10-20

**Authors:** Tomoya Ishida, Yuta Koshino, Masanori Yamanaka, Ryo Ueno, Shohei Taniguchi, Mina Samukawa, Hiroshi Saito, Hisashi Matsumoto, Yoshimitsu Aoki, Harukazu Tohyama

**Affiliations:** 1 Faculty of Health Sciences, Hokkaido University, Kita 12, Nshi 5, Kita-ku, Sapporo, 060-0812 Japan; 2 Faculty of Health Science, Hokkaido Chitose College of Rehabilitation, Satomi 2-10, Chitose, 066-0055 Japan; 3 Department of Rehabilitation, Hokushin Orthopaedic Hospital, Kikusui-motomachi 3-jo 3-chome 1-18, Sapporo, 003-0823 Japan; 4 Department of Orthopaedic Surgery, Hokushin Orthopaedic Hospital, Kikusui-motomachi 3-jo 3-chome 1-18, Sapporo, 003-0823 Japan

**Keywords:** Anterior cruciate ligament, Biomechanics, Risk factor, Prevention, Knee injury, Sex difference

## Abstract

**Background:**

A double-leg landing with or without a subsequent jump is commonly used to evaluate the neuromuscular control of knee abduction. However, the differences in frontal plane knee biomechanics between landings with and without a subsequent jump are not well known. The purpose of the present study was to investigate the effects of a subsequent jump on knee abduction, including during the early landing phase, in female and male subjects.

**Methods:**

Twenty-one female subjects and 21 male subjects participated. All subjects performed drop landing task (a landing without a subsequent jump) and drop vertical jump task (a landing with a subsequent jump). The subjects landed from a 30-cm height. In drop vertical jump, the subjects also performed a maximum vertical jump immediately after landing. The knee abduction angle and moment were analyzed using a 3D motion analysis system. A two-way analysis of variance (task × time) was performed to examine the effects of a subsequent jump on the knee abduction angle during the early landing phase in female and male subjects. Another two-way analysis of variance (task × sex) was performed to compare peak knee abduction angles and moments.

**Results:**

In female subjects, the knee abduction angle was significantly greater during drop vertical jump than during drop landing, as measured 45 to 80 ms after initial contact (*P* < 0.05). Significant task-dependent effects in the peak knee abduction angle (*P* = 0.001) and the abduction moment (*P* = 0.029) were detected. The peak knee abduction angle and the abduction moment were greater during drop vertical jump than during drop landing.

**Conclusions:**

Subsequent jumps cause greater knee abduction during the early landing phase only in female subjects. This finding may relate to the sex discrepancy in non-contact anterior cruciate ligament injuries. Additionally, the presence of a subsequent jump significantly increases the peak knee abduction angle and the peak knee abduction moment during landings. Therefore, compared with a landing task without a subsequent jump (drop landing), a landing task with a subsequent jump (drop vertical jump) may be advantageous for screening for knee abduction control, especially in female athletes.

## Background

Anterior cruciate ligament (ACL) injuries occur frequently in non-contact situations, such as landing from a jump, cutting, or pivoting [1–3]. Female athletes are at a higher risk of a non-contact ACL injury than male athletes [4]. Although ACL prevention programs that target female athletes do have a preventive effect [5–7], the overall number of ACL injuries in female athletes remains high [4]. One of the reasons for the continued elevated incidence of female ACL injury is that prevention programs have not spread effectively to athletes or coaches [8]. It is therefore important that preventive interventions emphasize those with a higher risk of ACL injury due to modifiable factors [8].

Hewett et al. [9] found that the peak knee abduction angle and the peak abduction moment during a landing were significant predictors of ACL injury. Video analysis studies of ACL injuries showed that valgus collapse occurs in most female cases [2, 10–12]. ACL injuries were believed to occur immediately after ground contact [11–13], and one study suggested that a rapid increase in the knee abduction angle during the early landing phase was a mechanism behind ACL injury [11]. Therefore, evaluating knee abduction control during the early landing phase as well as the peak knee abduction angle and the knee abduction moment during landing is important.

Since a double-leg landing is one of the most frequent causes of an ACL injury [12], landings with or without a subsequent jump are commonly used to evaluate the neuromuscular control of knee abduction [9, 14–18]. However, the differences in frontal plane knee biomechanics between landings with and without a subsequent jump remain poorly characterized. A previous study that directly compared landing tasks with and without a subsequent jump could not detect a difference in the knee abduction moment or the abduction angle at peak tibial shear forces [15]. Bates et al. [14] observed a significant difference in the peak knee abduction moment and the abduction angle between the 1st and 2nd landings of a drop vertical jump task, but the two landings involved different horizontal movements before landing. In addition, no study has examined the effects of a subsequent jump on knee abduction during the early landing phase. Understanding the effects of a subsequent jump on frontal plane knee biomechanics, including during the early landing phase, would be informative for clinicians as they evaluate knee abduction control. Furthermore, although the effects of a subsequent jump on sagittal plane biomechanics during a landing were found to differ between females and males [19], it remains unknown whether changes in frontal plane knee biomechanics also differ between females and males. Since sagittal plane hip and knee kinematics relate to frontal plane knee biomechanics during a landing [18], the effects of a subsequent jump on frontal plane knee biomechanics may differ between female and male subjects. Investigating sex differences in the effects of a subsequent jump on frontal plane knee biomechanics during a landing would be helpful in understanding the sex discrepancy in the incidence of ACL injuries.

The purpose of the present study was to investigate the effects of a subsequent jump after a landing on knee biomechanics, especially for the frontal plane, including during the early landing phase, in female and male subjects. The hypotheses of the study are that the knee abduction angle and the abduction moment are greater during landings followed by a jump than during landings without a subsequent jump and that the effects of a subsequent jump on knee abduction differ between female and male subjects.

## Methods

### Subjects

Twenty-one female subjects (mean ± SD: age 21.3 ± 1.2 years; height 161.5 ± 6.6 cm; mass 54.5 ± 7.8 kg) and 21 male subjects (age 21.4 ± 1.7 years; height 173.4 ± 5.2 cm; mass 63.2 ± 8.1 kg) participated in this study. All subjects had experience with regular sports activities, such as basketball, soccer, and handball. Subjects were excluded from this study if they reported any history of a musculoskeletal injury within the prior 6 months, a fracture or surgery of the lower extremities or trunk, or knee injuries, or had previously participated in jump/landing training or an ACL prevention program. Institutional review board approval and informed consent were obtained before this study was performed.

### Procedures and data collection

**Fig. 1:**
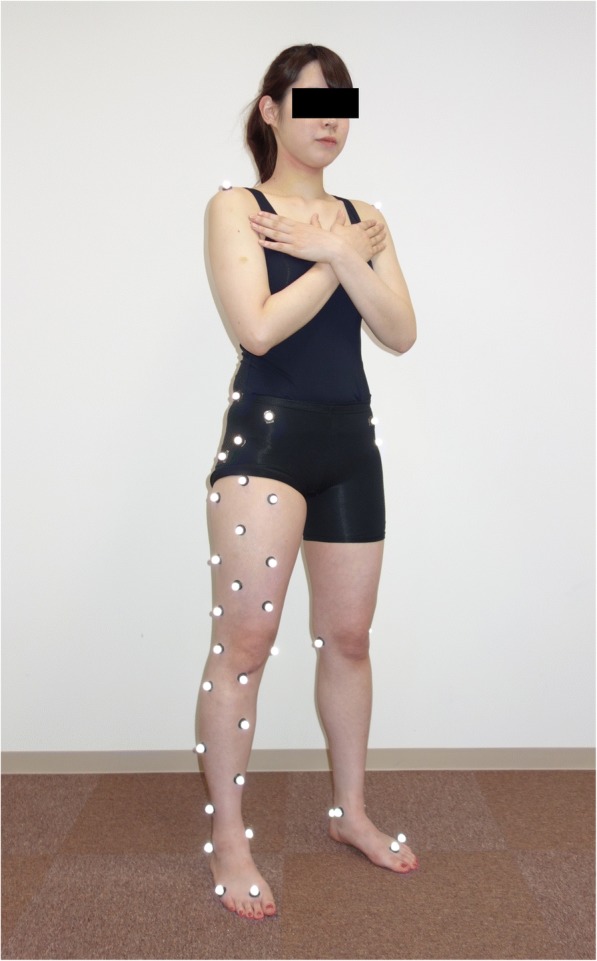
Marker placement

**Fig. 2:**
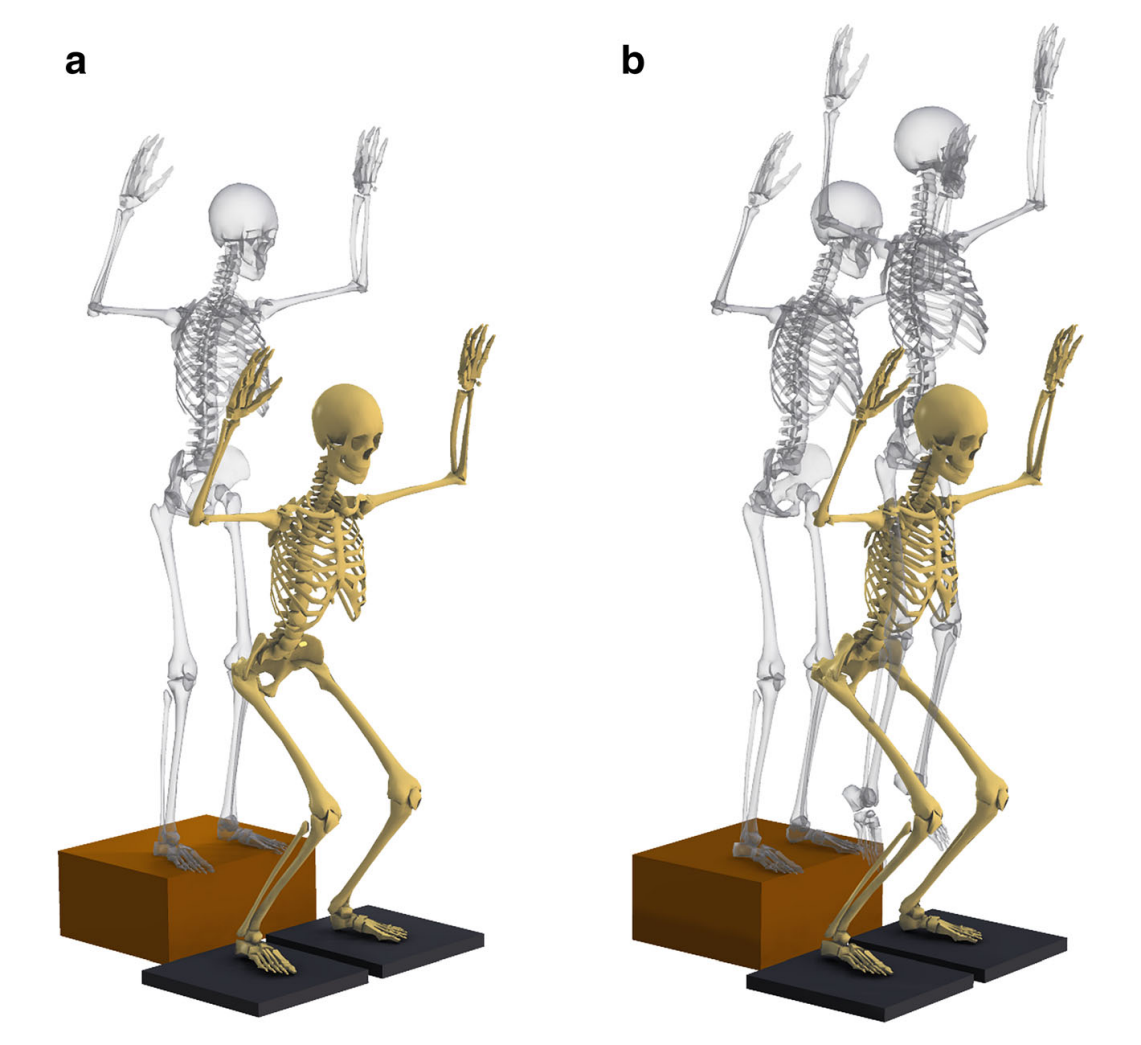
The two landing tasks. In drop landing (DL, landings without a subsequent jump), the subjects drop off a 30-cm high box and land on the force plates (**a**). During drop vertical jump (DVJ, landings with a subsequent jump), the subjects drop off the box and then jump immediately after landing (**b**)

The right legs of all subjects were analyzed because the dominant leg, which was defined as the leg used for kicking a ball, was the right one in all subjects. Retroreflective markers were placed on the sacrum and right iliac crest as well as the bilateral shoulders, anterosuperior iliac spines, greater trochanters, hips, medial and lateral knees, medial and lateral ankles, heels, second and fifth metatarsal heads, and right thigh and shank clusters of each subject who stood with bare feet (Fig. 1). Static standing trial data were then collected for each subject to obtain a zero reference. After the collection of zero-reference data, the subjects performed landings with or without a subsequent jump in random order. During landings without a subsequent jump (drop landing, DL), the subjects stood with their feet shoulder-width apart on a box that was 30 cm high [15]. The subjects then dropped off the box and landed on two force plates, one for each foot (Fig. 2a). During landings with a subsequent jump (drop vertical jump, DVJ), the subjects stood on the box, dropped off the box, and landed on the two force plates in the same way as in the task without a subsequent jump, but then performed a maximum vertical jump immediately after landing [9, 15] (Fig. 2b). The first landing from the box was analyzed. Through the two landing tasks, the subjects were asked to keep their hands at head level.

All data were collected with EVaRT 4.4 (Motion Analysis Corporation, Santa Rosa, CA, USA) using a motion analysis system with six high-speed digital cameras (Hawk cameras, Motion Analysis Corporation) and synchronized force data (Type 9286, Kistler AG, Winterthur, Switzerland). The sampling rates were set at 200 Hz for camera data and at 1000 Hz for force data. The camera position was considered and standardized before testing to reduce marker-trajectory gaps less than 20 frames (10% of the capture frequency), as a previous study deemed good in trials [20].

### Data processing and reduction

Marker-trajectory gaps were filled based on the coordinate data of three other markers in the same segment. The kinematics of the knee joint were calculated using a joint coordinate system and global optimization techniques with the SIMM 6.0.2 software (Musculo Graphics, Santa Rosa, CA, USA) [21]. The knee joint center was defined as the midpoint of the medial and lateral epicondyle markers. The hip join center was identified based on a previous study [22]. Zero references were set at specific knee angles during standardized static standing [23]. The external knee abduction and flexion moments were calculated using the inverse dynamics technique and normalized to each subject’s body weight and height (Nm/(kg*m)) using a custom MATLAB program (MathWorks, Inc., Natick, MA, USA). The initial ground contact (IC) was defined as the time when the vertical ground reaction force exceeded 10 N [23]. The knee abduction and flexion angles were analyzed between 0 and 80 ms after IC, since an ACL injury is thought to usually occur within approximately 80 ms after IC [11]. The peak knee abduction and flexion moments from IC to peak knee flexion were also calculated.

### Statistical analysis

Intraclass correlation coefficients (ICCs) were calculated as within-session reliability (ICC (3, *k*)) [23]. The ICC classifications were based on Fleiss (< 0.4 was poor, 0.4 to 0.75 was fair to good, > 0.75 was excellent) [24]. The typical errors that are reported in the measurement units were also calculated [23, 25].

A two-factor repeated measures analysis of variance (ANOVA) was used to examine the effects of subsequent jumps and time on knee abduction and flexion motions in the 80 ms after IC in female and male subjects. A mixed-model ANOVA was used to examine the effects of subsequent jumps and sex differences on the peak knee angle and moment. Bonferroni tests were used for post hoc comparisons. The statistical significance level was set at *P* < 0.05. These statistical analyses were performed using the IBM SPSS Statistics 22 software program (IBM, Armonk, NY, USA).

## Results

The ICC values of knee abduction angle during the early landing phase were greater than 0.951 and were classified as excellent for both landing tasks. The average typical errors for DVJ and DL were 0.9 ± 0.1° (range 0.7–1.1°) and 0.8 ± 0.1° (range 0.6–0.9°). Concerning knee flexion angle during the early landing phase, the ICCs were greater than 0.941 and were classified as excellent for both landing tasks. The average typical errors for DVJ and DL were 1.7 ± 0.3° (range 1.4–2.3°) and 1.7 ± 0.2° (range 1.4–1.9°). The ICCs and typical errors for peak angles and peak moments are presented in Table 1. All ICCs for these discrete data were classified as excellent.

**Table 1:** Within-session reliability of discrete data

Variable	DVJ (landing with a subsequent jump)		DL (landing without a subsequent jump)	
	ICC (3, *k*)	Typical error	ICC (3, *k*)	Typical error
Peak joint angles (°)				
Knee abduction	0.988	0.8	0.986	0.7
Knee flexion	0.961	2.8	0.980	2.6
Peak joint moments (Nm/(kg*m))				
Knee abduction	0.890	0.05	0.811	0.06
Knee flexion	0.947	0.07	0.889	0.06

**Fig. 3:**
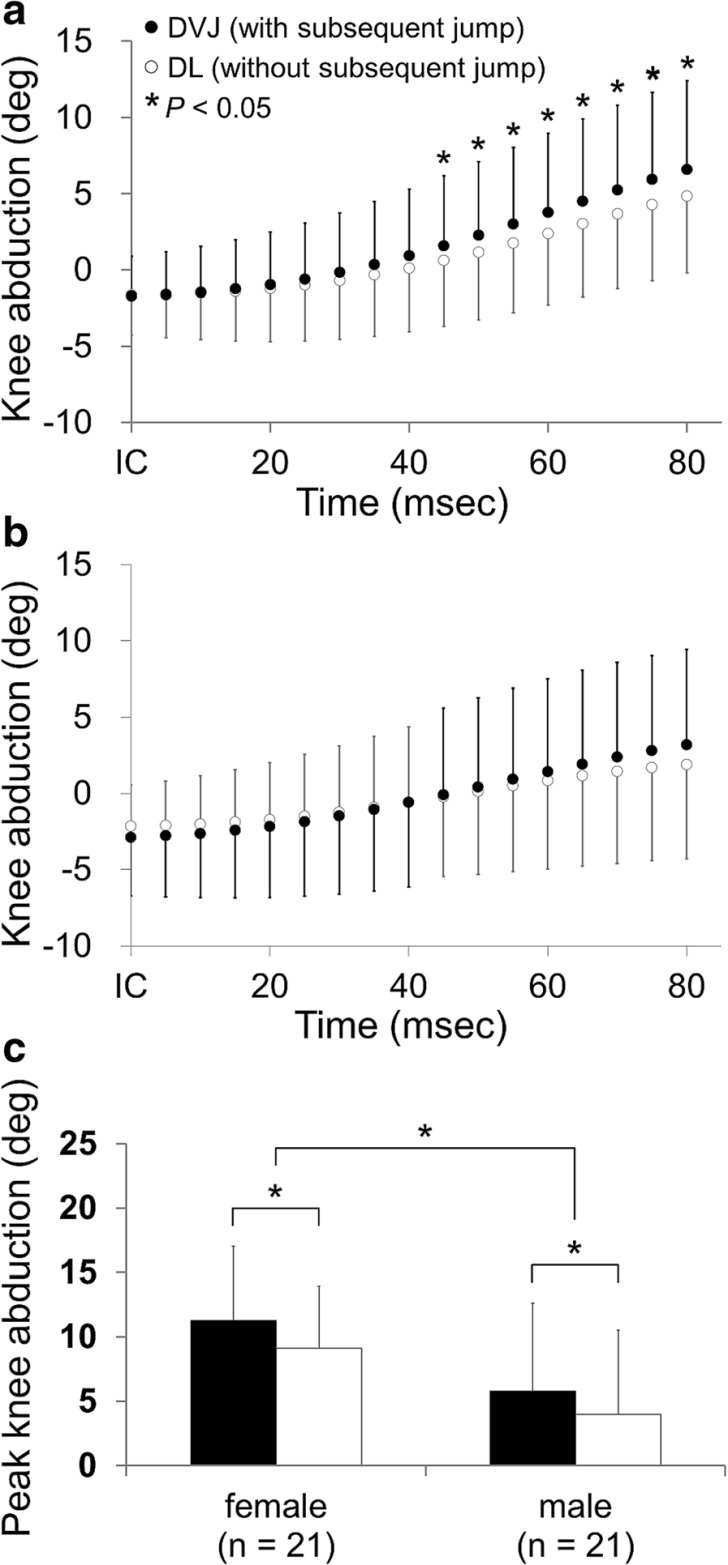
Knee abduction angles during the early landing phase for female (**a**) and male subjects (**b**) and peak knee abduction angles during the landing phase (**c**). *indicates a significant difference (*P* < 0.05). IC, initial contact

For female subjects, the knee abduction angle was significantly greater during DVJ (landings with a subsequent jump) than during DL (landings without a subsequent jump), as measured 45 to 80 ms after IC (*P* = 0.002–0.027, power = 0.62–0.94) (Table 2 and Fig. 3a). The results of a two-way ANOVA demonstrated the significant impact of time and subsequent jumps as well as their interaction in female subjects (time effect: *P* < 0.001, subsequent jump effect: *P* = 0.024, interaction: *P* < 0.001). In contrast, there were no significant differences in knee abduction angle between DVJ and DL in male subjects (*P* = 0.059–0.996) (Table 2 and Fig. 3b). The 2-way ANOVA demonstrated the significant effects of time and the time-by-subsequent jump interaction (both: *P* < 0.001). A subsequent jump did not have a significant effect on the knee abduction angle of male subjects (*P* = 0.838). With respect to the peak knee abduction angle during landing, significant effects of sex and a subsequent jump were found (sex effect: *P* = 0.005, subsequent jumping effect: *P* = 0.001), while there was no interaction between sex and a subsequent jump (*P* = 0.741) (Fig. 3c). Female subjects demonstrated greater peak knee abduction angle than male subjects (95% CI 1.7–8.9°). The peak knee abduction angle was significantly greater during DVJ (landings with a subsequent jumps) than DL (landings without a subsequent jump) (95% CI 0.9–3.1°).

**Table 2:** The differences in knee joint angle between DVJ (landings with a subsequent jump) and DL (landings without a subsequent jump)

Time^a^	Abduction angle (°)		Flexion angle (°)	
	Female	male	female	male
0 ms	− 0.1 (− 0.5 to 0.3)	−0.7 (− 1.8 to 0.3)	**5.0 (3.3 to 6.6)**	**9.4 (5.8 to 13.0)**
5 ms	0.0 (−0.5 to 0.4)	− 0.7 (− 1.7 to 0.3)	**5.2 (3.5 to 6.8)**	**9.4 (5.9 to 13.0)**
10 ms	0.0 (− 0.5 to 0.6)	− 0.6 (− 1.7 to 0.4)	**5.3 (3.6 to 7.0)**	**9.5 (6.0 to 13.1)**
15 ms	0.1 (− 0.4 to 0.7)	− 0.5 (− 1.6 to 0.5)	**5.5 (3.7 to 7.2)**	**9.6 (6.1 to 13.1)**
20 ms	0.3 (− 0.4 to 0.9)	− 0.5 (− 1.5 to 0.6)	**5.6 (3.8 to 7.3)**	**9.7 (6.2 to 13.2)**
25 ms	0.4 (− 0.3 to 1.1)	− 0.4 (− 1.4 to 0.7)	**5.7 (3.9 to 7.4)**	**9.8 (6.3 to 13.3)**
30 ms	0.5 (− 0.2 to 1.3)	− 0.2 (− 1.3 to 0.8)	**5.7 (3.9 to 7.5)**	**9.9 (6.4 to 13.4)**
35 ms	0.7 (− 0.1 to 1.5)	− 0.1 (− 1.2 to 0.9)	**5.7 (3.9 to 7.5)**	**10.0 (6.6 to 13.5)**
40 ms	0.0 (0.0 to 1.7)	0.0 (−1.1 to 1.1)	**5.7 (3.9 to 7.5)**	**10.2 (6.7 to 13.6)**
45 ms	**1.0 (0.1 to 1.8)**	0.1 (−1.0 to 1.2)	**5.7 (3.9 to 7.5)**	**10.2 (6.8 to 13.7)**
50 ms	**1.1 (0.2 to 2.0)**	0.3 (−0.9 to 1.4)	**5.6 (3.8 to 7.5)**	**10.3 (6.9 to 13.8)**
55 ms	**1.3 (0.4 to 2.1)**	0.4 (−0.8 to 1.6)	**5.6 (3.7 to 7.4)**	**10.4 (6.9 to 13.8)**
60 ms	**1.4 (0.5 to 2.3)**	0.6 (−0.6 to 1.8)	**5.5 (3.6 to 7.4)**	**10.4 (6.9 to 13.9)**
65 ms	**1.5 (0.5 to 2.4)**	0.8 (−0.5 to 2.0)	**5.4 (3.4 to 7.3)**	**10.4 (6.9 to 13.9)**
70 ms	**1.6 (0.6 to 2.5)**	0.9 (−0.3 to 2.2)	**5.3 (3.2 to 7.3)**	**10.4 (6.9 to 13.9)**
75 ms	**1.7 (0.7 to 2.7)**	1.1 (−0.2 to 2.4)	**5.1 (3.0 to 7.2)**	**10.4 (6.8 to 13.9)**
80 ms	**1.7 (0.7 to 2.7)**	1.3 (−0.1 to 2.6)	**5.0 (2.8 to 7.2)**	**10.3 (6.7 to 14.0)**

Data are presented as means (95% confidence intervals)

Positive values indicate that the angles during DVJ were greater than those during DL

Boldface indicates significant differences between DVJ and DL

^a^Time 0 indicates initial contact

**Fig. 4:**
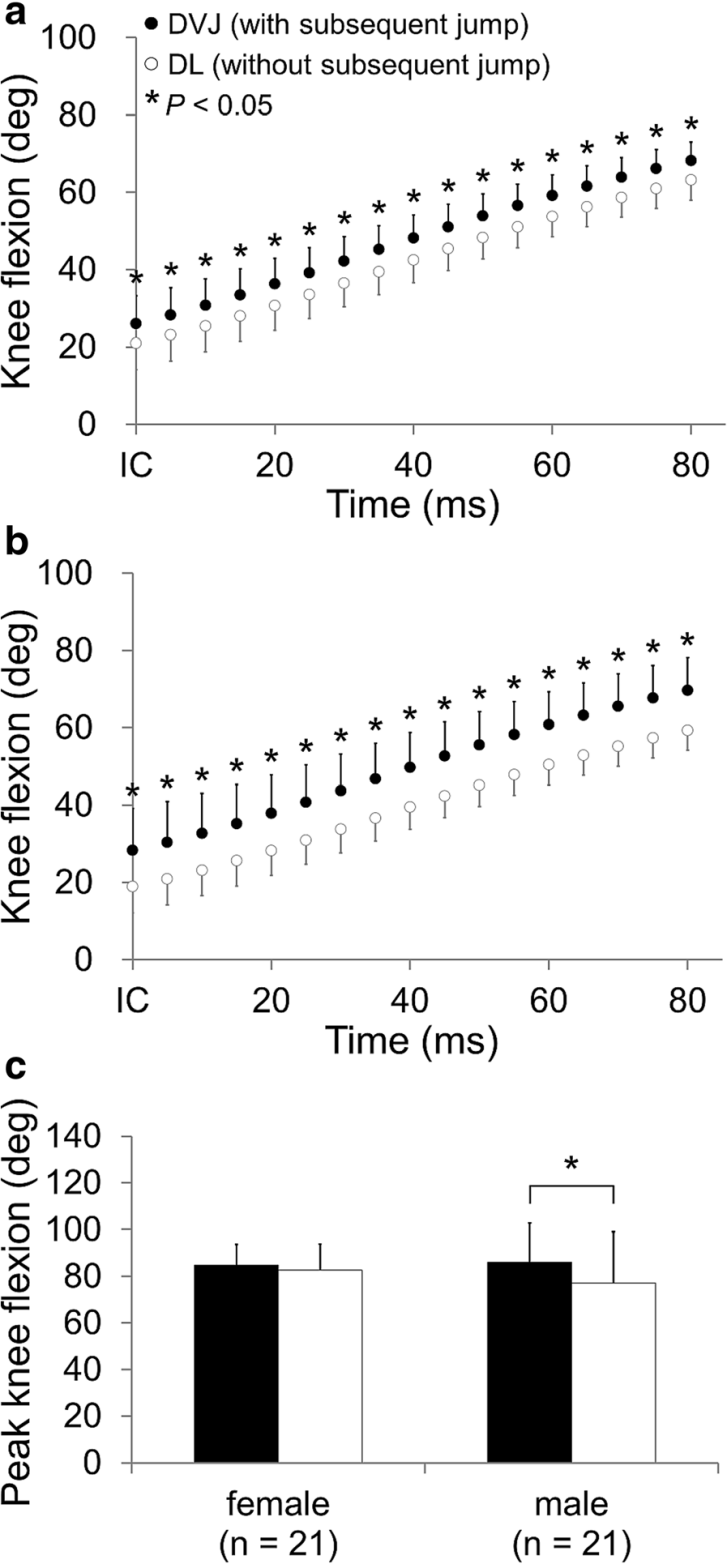
Knee flexion angles during the early landing phase for female (**a**) and male subjects (**b**) and the peak knee abduction angle measured during the landing phase (**c**). *indicates a significant difference (*P* < 0.05). IC, initial contact

The knee flexion angles in female and male subjects were significantly greater during DVJ (landings with a subsequent jump) than during DL (landings without a subsequent jump), as measured 0 to 80 ms after IC (female: all *P* < 0.001, power > 0.99; male: all *P* < 0.001, power > 0.99) (Table 2 and Fig. 4). The 2-way ANOVA demonstrated the significant effects of time and a subsequent jump on the knee flexion angles of both female and male subjects (all *P* < 0.001), while no interaction effect was observed (female: *P* = 0.654, male: *P* = 0.227). There was no significant difference in the peak knee flexion angle between DVJ and DL in female subjects (*P* = 0.446, 95% CI -3.7–8.2°), while male subjects had a significantly greater angle during DVJ (landings with a subsequent jump) than during DL (landings without a subsequent jump) (*P* = 0.004, power = 0.70, 95% CI 3.0–14.9°) (Fig. 4c). The 2-way ANOVA demonstrated the significant effect of a subsequent jump on the peak knee flexion angle (*P* < 0.001). In contrast, sex did not have a significant effect, and there was no interaction between these factors (sex effect: *P* = 0.622, interaction: *P* = 0.113).

**Fig. 5:**
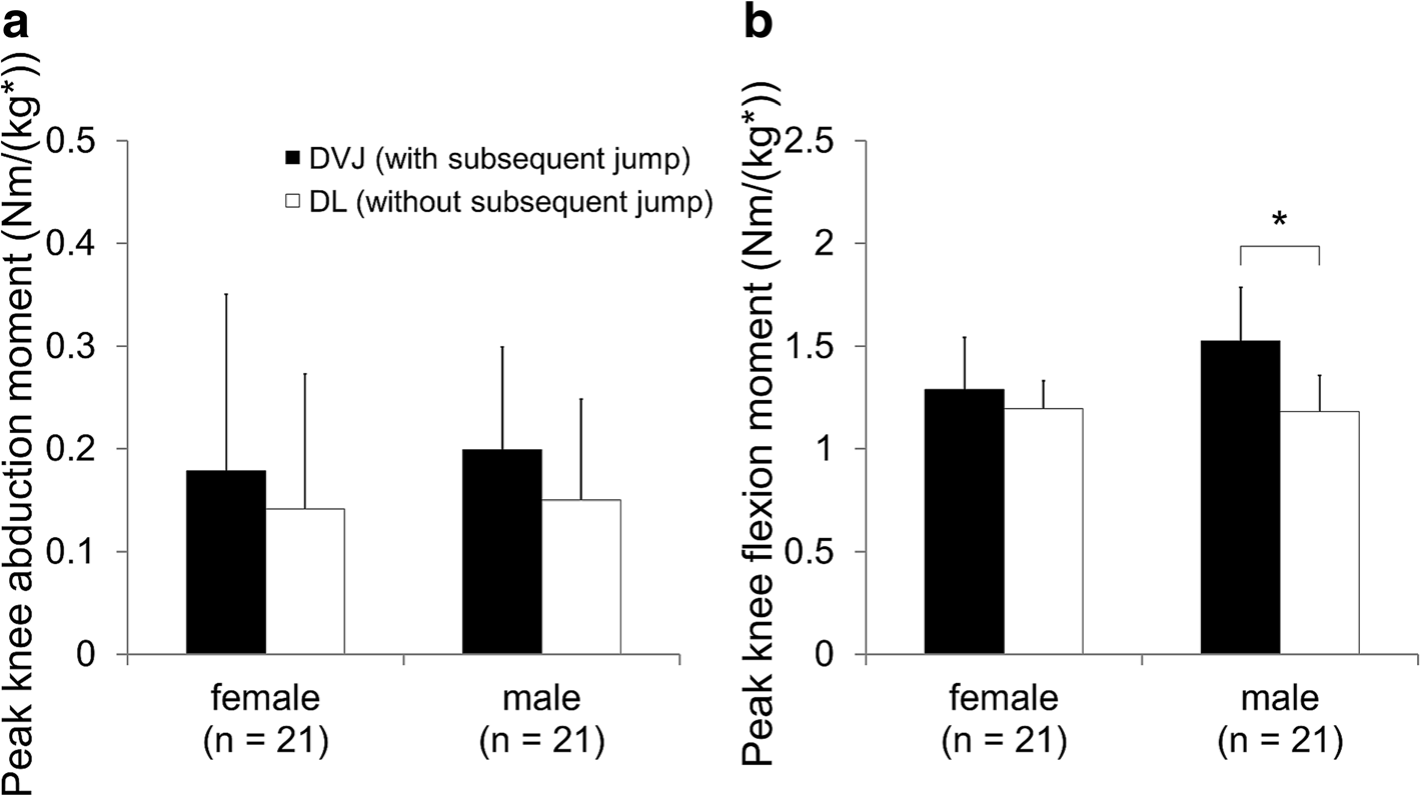
Peak knee abduction (**a**) and flexion moments (**b**). *indicates a significant difference between DVJ and DL, as detected by the post hoc test (*P* < 0.05)

With respect to the peak knee abduction moment, we could not find a statistically significant difference between DVJ and DL in either female or male subjects (female: *P* = 0.179, male: *P* = 0.073) (Fig. 5a). There were no significant differences in the peak knee abduction moment between female and male subjects (DVJ: *P* = 0.636, DL: *P* = 0.824). The ANOVA demonstrated that a subsequent jump had a significant impact on the peak knee abduction moment (*P* = 0.029, 95% CI 0.05–0.081 Nm/(kg*m)). In contrast, sex did not have a significant effect, and there was no interaction between these factors (sex effect: *P* = 0.684, interaction: *P* = 0.738). The peak knee flexion moment in male subjects was significantly greater during DVJ (landings with a subsequent jump) than during DL (landings without a subsequent jump) (*P* < 0.001, power > 0.99, 95% CI 0.245–0.446 Nm/(kg*m)) (Fig. 5b), although we failed to detect a significant difference in female subjects (*P* = 0.067, 95% CI -0.007–0.194 Nm/(kg*m)). The 2-way ANOVA also found significant effects of the subsequent jump and an interaction with the peak knee flexion moment (subsequent jumping effect: *P* < 0.001, interaction: *P* = 0.001), while we could not detect a significant impact of sex on the peak knee flexion moment (*P* = 0.052).

## Discussion

The purpose of this study was to clarify the effects of a subsequent jump on knee biomechanics, especially for the frontal plane. The results showed that the knee abduction angle was significantly greater during DVJ (landings with a subsequent jump), as measured 45 to 80 ms after IC, but only in female subjects. In addition, the significant main effects of a subsequent jump on the peak knee abduction angle and the peak abduction moment were confirmed. These findings suggest that a subsequent jump after a landing impacts frontal plane knee biomechanics during the landing, especially in female subjects.

A video analysis study of ACL injury cases suggested that ACL injuries occur within 80 ms after landing and that the knee abduction angle increased rapidly during the early landing phase [11]. Cadaveric simulation studies suggest that a knee abduction load with an anterior tibial shear force during the early landing phase plays a crucial role in ACL injury [13, 26–28]. The present study showed that the knee abduction angle of female subjects was significantly greater during DVJ (landings with a subsequent jump) than during DL (landings without a subsequent jump), as measured 45 to 80 ms after IC. The differences between the two landing tasks were small, but within-session reliability was classified as excellent, and those differences exceeded within-session typical errors. In contrast, male subjects did not demonstrate a significant difference in knee abduction angle within 80 ms after IC between the two landing tasks. The different effects of a subsequent jump on the knee abduction angle during the early landing phase between female and male subjects may relate to the sex discrepancy in the incidence of ACL injuries [4].

The results of this work support those of a previous study that measured significant differences in the peak knee abduction angle and abduction moment between the 1st and 2nd landings of DVJ [14]. Since the subjects dropped from the same height in both landing tasks, the differences in the peak knee abduction angle and abduction moment were thought to be caused by the subject’s preparation for generating the power required to perform a subsequent jump. The literature has shown that the presence of a subsequent jump also affected lower extremity muscle activities before and after a landing [29]. These muscle activity changes may affect frontal plane knee biomechanics during the landing. Further studies are needed to investigate muscle activity and knee biomechanics to better understand the mechanism that underlies the effects caused by a subsequent jump. Additionally, to the best of our knowledge, it remains unknown what role frontal plane knee biomechanics plays in plyometric jumping performance. Understanding how frontal plane knee biomechanics, including power or work calculations, contributes to plyometric jumping performance may help explain the mechanism that underlies the effects of a subsequent jump on frontal plane knee biomechanics during a landing.

We found that male subjects had a significantly greater peak knee flexion moment during DVJ (landings with a subsequent jump) than during DL (landings without a jump). A greater knee flexion moment reflects a greater quadriceps force during a landing task [30], and quadriceps contraction resists a knee valgus moment [31]. Male subjects may have increased frontal plane knee stability with a greater quadriceps force. In addition, previous studies have shown that females have greater laxity, or less stiffness, during knee valgus rotation than males [32–34]. These neuromuscular and structural differences may be reasons for the observed differences in the impact of a subsequent jump on the knee abduction angle during the early landing phase between female and male subjects.

With respect to clinical relevance, the present study shows that a landing task with a subsequent jump can result in greater knee abduction compared with landings without a subsequent jump, especially in females. Hewett et al. [9] reported that greater knee abduction moment and angle during a DVJ task were the significant predictors of ACL injury. Clinicians should use a landing task with a subsequent jump, e.g., DVJ, to evaluate knee abduction control. Additionally, ACL prevention programs should include landings with a jump as well as landings without a jump, in line with previous successful programs [5–7].

This study has some limitations. We examined double-leg landing tasks, while single-leg landing tasks with or without a subsequent jump are also commonly used to evaluate knee biomechanics. The effects of a subsequent jump after a single-leg landing may differ from the effects observed after a double-leg landing. Second, the present study did not investigate the mechanisms that underlie the effects of a subsequent jump on frontal plane knee biomechanics. If this mechanism is understood, an intervention may be developed. Future studies should include analyses of muscle activity and lower-extremity jumping mechanics.

## Conclusions

The present study showed that a jump performed immediately after a landing significantly increases the knee abduction angle during the early landing phase in female subjects but not in males. These findings may relate to the known sex discrepancies in the incidence of non-contact ACL injuries. Additionally, the presence of a subsequent jump significantly impacts the subject’s peak knee abduction angle and abduction moment during a landing. A landing task with a subsequent jump, e.g., DVJ, is advantageous for evaluating knee abduction control in comparison with landings without a subsequent jump, especially in female subjects.

## Abbreviations

ACL: Anterior cruciate ligament
DL: Drop landing
DVJ: Drop vertical jump
IC: Initial contact
